# Study of Optical Properties of Surface Layers Produced by Laser Surface Melting and Laser Surface Nitriding of Titanium Alloy

**DOI:** 10.3390/ma12193112

**Published:** 2019-09-24

**Authors:** Aleksander Lisiecki

**Affiliations:** Silesian University of Technology, Faculty of Mechanical Engineering, Department of Welding Engineering, Konarskiego 18A street, 44-100 Gliwice, Poland; aleksander.lisiecki@polsl.pl

**Keywords:** titanium nitrides, reflectivity, absorption, high power diode laser

## Abstract

This study measured optical properties, such as specular, diffuse, and total reflection for 808 nm wavelength, characteristic for high power diode lasers radiation, from the surface of titanium alloy Ti6Al4V at delivery conditions, polished, and oxidized. Moreover, the optical properties of surface layers produced by high power direct diode laser (HPDDL) melting and nitriding were determined. Additionally, a methodology for determining the value of absorption for 808 nm wavelength of the HPDDL radiation on the surface of a melt pool during laser surface melting and nitriding of titanium alloy was proposed. The results show that the distinct differences in absorption affect the heat transfer, thermal conditions of laser heating and thereby the penetration depth during laser melting and nitriding of the titanium alloy.

## 1. Introduction

Titanium alloys have excellent mechanical properties, but during the manufacture of machinery parts, components or tools it is often necessary to apply additional surface treatments to improve the tribological characteristic of working surfaces [1,2,3,4,5,6,7,8,9,10,11]. One of the methods of improving properties is laser gas nitriding (LGN) of titanium and its alloys. Laser gas nitriding involves heating or surface melting the titanium substrate in a nitrogen or nitrogen-rich atmosphere. Laser gas nitriding is a very efficient and flexible method for shaping the surface characteristics of commercially pure titanium and technical titanium alloys [1,2,3,4,5,6,7,8,9,10,11]. This is because of the large chemical affinity of titanium for nitrogen at elevated temperatures, particularly in a liquid state. Titanium has a strong tendency to absorb nitrogen at temperatures above 600 °C and precipitate nitrides ε-Ti_2_N, even at a relatively low nitrogen content in the solid solution (approx. 4 at. % of nitrogen at temp. 500 °C), and also δ-TiN at a higher nitrogen content (23.0 at. % of nitrogen at 1050 °C). Due to this feature, a composite surface layer with in situ synthesized nitrides, embedded in the metallic matrix of titanium, can be formed during laser gas nitriding of titanium and titanium alloys [5,6,7,8,9,10,11]. Additionally, nitrogen has the effect of strengthening the structure, as an interstitial element, which distorts the lattice by occupying the available interstitial sites in the structure [7,11]. These types of titanium matrix composite (TMC) surface layers have significantly higher hardness and exhibit higher resistance to abrasive wear than the substrate of pure titanium or conventional technical titanium alloys [8,9,11].

So far, the process of titanium nitriding has been carried out mainly by solid state Nd:YAG (Yttrium-Aluminum Garnet doped with neodymium ions) and gaseous CO_2_ lasers that emit radiation with a wavelength of 1.06 and 10.6 µm, respectively [7,8,9,10,11,12,13,14,15,16,17,18,19,20,21]. The next generation of lasers recently introduced to the industry are high power diode lasers. These lasers show some advantages in the field of laser surface treatment, compared to the above mentioned solid state and gaseous lasers [7,10,22,23]. Diode lasers provide a favorable rectangular shape of the laser beam spot, and uniform energy distribution across the spot, and also are characterized by a short wavelength in the very near infrared range from 0.808 to 0.960 µm, which is advantageous because of the relatively high absorptivity on the surface of most metals, particularly as compared to the gaseous CO_2_ lasers with a wavelength of 10.6 µm [11]. However, to ensure stable and reproducible processing conditions, a precise control of heat input during laser processing is required. The “heat input” is often used as synonymous with the “energy input”. However, the energy input is defined as the amount of energy per unit of a length:

Ev = P/v (J/mm),(1)

where P is the power of the laser beam (W) and v is the scanning speed (mm/s).

In turn, the heat input defines the actual amount of heat transferred to the material, so it depends on the efficiency of heat transfer. In the case of heating the surface by a laser beam the efficiency of heat transfer is proportional to the surface absorption “A” of the laser radiation under such conditions. The surface absorption depends on many factors such as material type, surface roughness, surface temperature, wavelength of laser radiation, power density of laser beam, etc. Therefore, knowledge of the material surface absorption under different conditions is the necessary factor to ensure proper thermal conditions and control of the laser treatment process, and also it is necessary to develop a reliable numerical model of laser gas nitriding by the high power diode laser [24,25,26]. Knowledge of optical properties is also necessary for reliable non-contact temperature measurements by means of infrared cameras or pyrometers.

Previous studies, and the results published by other researches, also show that the penetration depth during laser melting of titanium and its alloys in a nitrogen atmosphere is significantly greater than in an inert gas atmosphere (e.g., in pure argon) [11,21,22]. This phenomenon has not yet been clearly elucidated. The key to explain this phenomenon is the analysis of thermal conditions during laser nitriding, efficiency of heat transfer and to determine the temperature gradients.

On the other hand, methods of non-contact temperature measuring during laser processing are commonly used for monitoring and controlling the process. In the case of non-contact temperature measuring of materials, the absolute value of temperature indicated by infrared camera or pyrometer depends strongly on the assumed value of the material emissivity at operating wavelength or in an operating spectral range [24,25,26]. Therefore, non-contact measurements of temperature require knowledge of the normal spectral emissivity **ε_n_** of the material at a given wavelength or range of wavelengths. Moreover, the value of spectral emissivity depends on the material surface conditions such as presence of oxides, thickness of the oxides’ layer, roughness, and temperature. Unfortunately, the literature data or materials databases usually provide just the information on hemispherical emissivity, in addition to surfaces of pure metallic or nonmetallic materials. Patsalas et al. developed an extensive review of the literature on the optical properties of TiN films grown by various techniques and on various substrates [24]. It provides wide data on optical reflectivity spectra of TiN at normal incidence but just at room temperature. In addition, the data reported by researchers differ significantly [24,25].

Therefore, the author attempts to determine optical properties such as emissivity, reflectivity, and absorption of surface layers produced on the substrate of titanium alloy Ti6Al4V by laser surface melting and nitriding using the high power direct diode laser emitting radiation with a wavelength of 808 nm.

In addition, the author attempts to estimate the absorption coefficients of the metal surface in a liquid state during laser melting of titanium alloy Ti6Al4V in a pure argon atmosphere and in a pure nitrogen atmosphere.

## 2. Materials and Methods

The aim of the first stage of the study was to determine and compare the optical properties of the titanium alloy Ti6Al4V (Grade 5 according to the standard ASTM B 265-99) with different surface conditions (delivery conditions, oxidized, and polished), as well as the surface layers produced by laser melting the titanium alloy in an argon atmosphere, and laser nitriding in a gaseous nitrogen atmosphere. The trials of laser surface melting and laser gas nitriding were conducted in a very wide range of processing parameters and technological conditions. The scanning speed varied from 200 to 800 mm/min as did the laser output power from 0.8 to 1.8 kW, thus the energy input was from 70 to 270 J/mm. Additionally, the trials of laser processing were at a constant energy input of 270 J/mm (400 mm/min and 1.8 kW) and different gaseous atmospheres (100% Ar, 80% Ar + 20% N_2_, 70% Ar + 30% N_2_, 60% Ar + 40% N_2_, and 50% Ar + 50% N_2_) were used (Figure 11). Therefore, representative samples with surface layers produced by laser melting in pure argon and pure nitrogen were selected for the study of optical properties. The selection criteria were an even surface with the lowest possible roughness.

Next, based on the results and findings of the first stage of the study, experiments were planned to determine the absorption for an 808 nm wavelength of high power diode laser radiation on the surface of a melt pool during laser surface melting and nitriding of titanium alloy Ti6Al4V.

These studies consisted of real-time measurements during laser melting or nitriding the titanium alloy substrate. Therefore, the processing parameters have been narrowed and adapted to the specificity of the temperature measurements in such a way as to ensure stability and repeatability of the results.

Real-time measurements were carried out at a constant scanning speed of 200 mm/min, different laser output power (determined precisely by setting the current supplying diode packages of the laser generator), and thus different energy input, starting with an energy input above 270 J/mm (Table 1).

The test surface layers for the study of optical properties, as well as for real-time measurements were produced as single stringer beads. All trials of laser melting and nitriding were carried out at the focal length of 82.0 mm and dimensions of the rectangular laser beam spot 1.8 × 6.8 mm. The focal plane was set at the top surface of the treated samples and the rectangular beam spot was positioned perpendicularly to the direction of scanning, in such a way that the spot width was 6.8 mm. To provide the proper and controlled gaseous atmosphere for laser processing, the specimens for study of optical properties were placed in a gas chamber filled up with argon or nitrogen or a mixture of argon and nitrogen of 99.999% purity. In turn, during the tests of real-time measurements the gas atmosphere was provided by a cylindrical nozzle with a diameter of 12.0 mm, and the gas flow rate was kept at 15.0 l/min.

**Figure 1:**
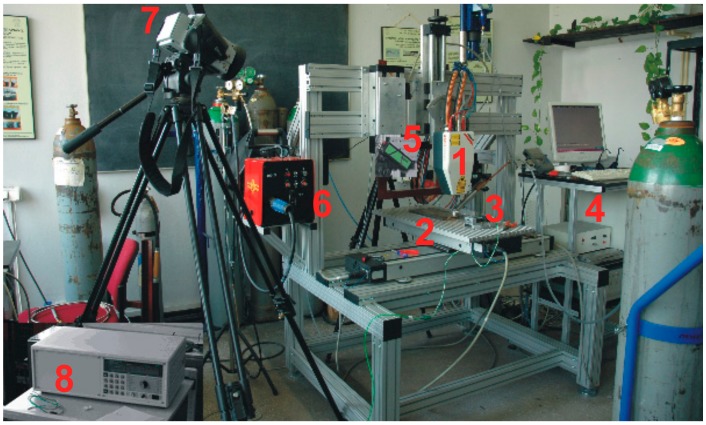
A view of experimental setup equipped with the high power direct diode laser (HPDDL) ROFIN SINAR DL020 (**1**), positioning system (**2**), mounting device (**3**), computer control system (**4**), pyrometer IMPAC IS 140 (**5**), electronic controller (**6**), infrared camera ThermaCAM P640 (**7**), and multichannel numerical recorder Agilent 34970A (**8**).

The high power direct diode laser (HPDDL) ROFIN DL 020 (ROFIN SINAR LASER, Hamburg, Germany) was applied in the study (Figure 1). This type of a laser emits the laser beam directly from the laser head, and it is called a “direct” laser. Additionally, the laser is characterized by a rectangular laser beam, uniform energy distribution across the beam spot, and a short wavelength. One of the characteristic features of this laser is its wavelength. Typically, it is given that the wavelength is 808 or 940 or even 960 nm. In fact, the emission spectral range of the specific HPDDL is relatively wide. The total emission spectral range is from 800 up to even 1000 nm but there are several dominant wavelengths such as 808, 940, and 960 nm. The most dominant is the 808 nm wavelength of the laser radiation, hence it is taken as the characteristic value for this type of an HPDDL.

**Figure 2:**
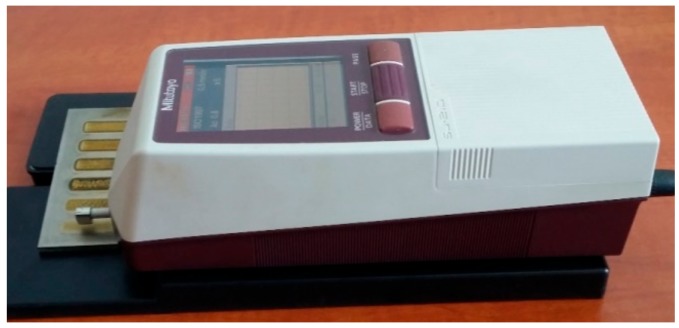
A view of the portable surface roughness tester SJ-210 Surftest Mitutoyo during measurements of roughness on the surface of stringer beads produced by laser gas nitriding of titanium alloy Ti6Al4V.

The properties of the tested surfaces were characterized by roughness and the thickness of the surface layer (oxide or titanium nitrides layer). Measurements of surface roughness were carried out by means of a portable surface roughness tester SJ-210 Surftest (Mitutoyo Corporation, Kanagawa, Japan) according to the standard ISO 4288 standards (Figure 2). The thickness of titanium nitrides or surface oxide layers were determined on microstructure images, obtained by a metallographic light microscope (LM) Nikon Eclipse MA100 (NIKON CORPORATION, Tokyo, Japan) and then by scanning electron microscopy (SEM) (Carl Zeiss, Oberkochen, Germany) with an energy dispersive spectrometer (EDS) (Carl Zeiss, Oberkochen, Germany).

**Figure 3:**
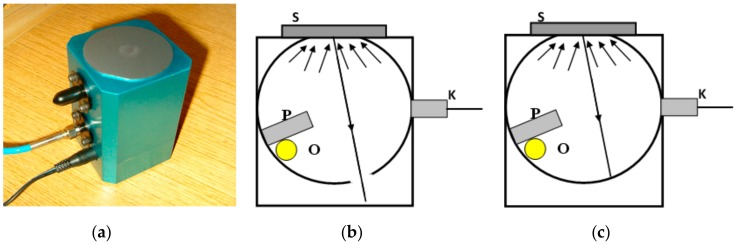
A view of the Ulbricht sphere applied for measurements of reflectance (**a**); and a scheme of the sphere design (O—source of white light, S—tested sample, P—aperture, K—fiber optics cable): (**b**) the aperture setting to measure the specular and diffuse reflections; (**c**) the aperture setting to measure the diffuse reflections only.

The measurements of reflectance of tested samples were performed by means of the Ocean Optics PC2000-ISA-PC Plug-in Spectrometer (Ocean Optics, Largo, FL, USA), ISP-REF Integrating Sphere, and WS-1 Diffuse Reflectance Standard. The main part of the measuring device is a so-called “Ulbricht sphere” ISP-REF Integrating Sphere, shown in Figure 3. The ISP-REF Integrating Sphere provides even surface illumination for reflectance measurements and also collects light to an optical fiber for emission experiments. The working wavelength range of the ISP-REF Integrating Sphere is 0.360–2.500 µm with the resolution ±0.5 nm. However, the scatter of results was significant in the measurement range wider than 0.450–1.130 µm, so it was decided to limit the measurement range to 0.500–1.100 µm.

The measurements were carried out at room temperature (about 20 °C) and both the specular and diffuse reflectivity were determined for each sample. The test samples with dimensions of 40 × 70 mm were prepared from a titanium alloy sheet with a thickness of 3.0 mm. Surfaces of the samples were prepared and treated in different ways. One surface was untreated in as-received conditions with a thin natural layer of oxides, one surface was polished, and another surface was strongly oxidized at 900 °C in air atmosphere. In addition, surface layers after laser melting in argon and nitrogen characterized by different surface topography and roughness were applied for measurements of reflectance.

The temperature of the solid and liquid surface of the titanium alloy during laser processing was measured by the pyrometer IMPAC IS 140 (IMPAC Infrared GmbH, Frankfurt, Germany) with a measurement range from 550–3300 °C and spectral range 0.7–1.1 µm.

**Figure 4:**
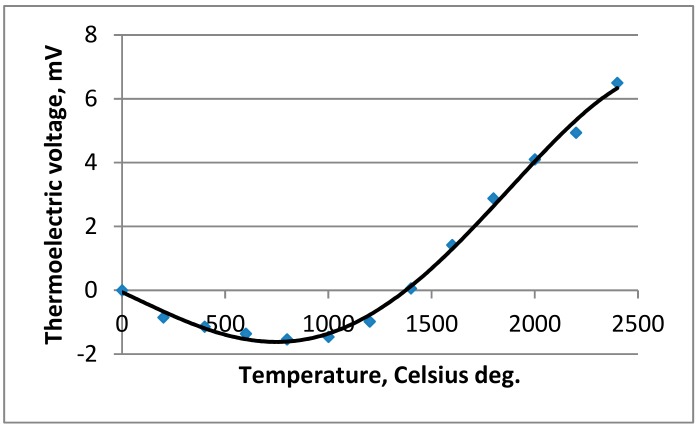
Characteristics of the non-standardized thermocouple W-Mo with a temperature range up to 2400 °C [27,28,29].

Additionally, high temperature, nonstandard thermocouple W-Mo with the measurement range up to 2400 °C, was used to calibrate the pyrometer. The characteristic of W-Mo thermocouple was determined based on the data provided by Caldwell, Michalski et al., and Blatt et al., as shown in Figure 4 [27,28,29].

**Figure 5:**
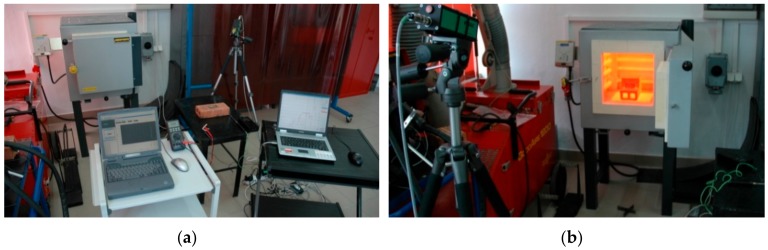
A view of experimental setup: (**a**) with an electronically controlled furnace and high temperature pyrometer IMPAC IS 140; (**b**) a view of temperature measurements on the surface titanium alloy sample in a high temperature range.

The multichannel numerical recorder Agilent 34970A (Agilent, Santa Clara, CA, USA) was applied for acquisition and recording of data. The procedure of calibration was performed on surfaces of as-received titanium alloy, on nitrided surface layers, and on a strongly oxidized surface. The samples were gradually heated in a protective gas atmosphere of argon inside an electronically controlled furnace (Figure 5).

**Figure 6:**
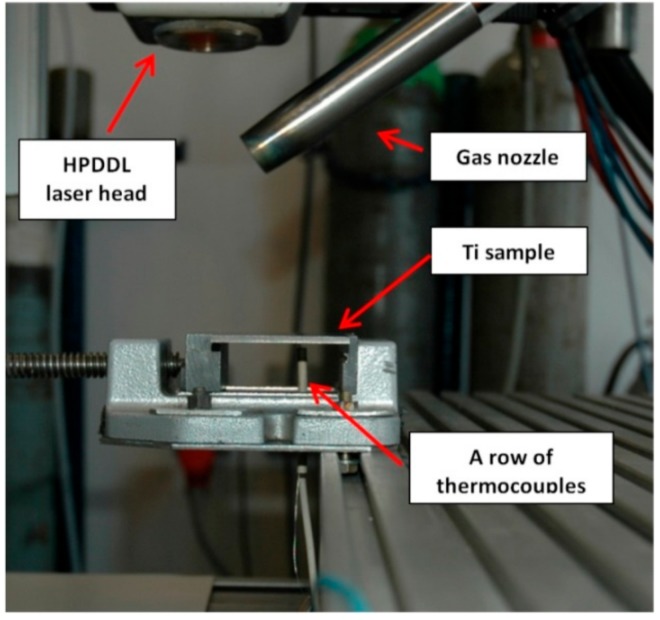
A view of the arrangement of non-standardized thermocouple W-Mo, mounted on the bottom surface of the sample used during the experimental study of heat conditions of HPDDL surface melting and nitriding.

During the gradual heating, temperature of the surface was measured simultaneously by the pyrometer and the thermocouple. Based on the temperature indications the values of emissivity in a spectral range of the IMPAC IS 140 were established for different surface conditions in a wide range of temperatures up to 1100 °C but below the melting point of titanium alloy. In the next step the temperature measurements of the liquid surface of the melt pool during laser melting were performed simultaneously by means of the pyrometer and the thermocouple (Figure 1 and Figure 6). The thermocouples were mounted in cylindrical holes 0.5 mm in diameter and 1.5 mm deep from the bottom side of the sample. The thermocouples were arranged in rows, as shown in Figure 6.

**Figure 7:**
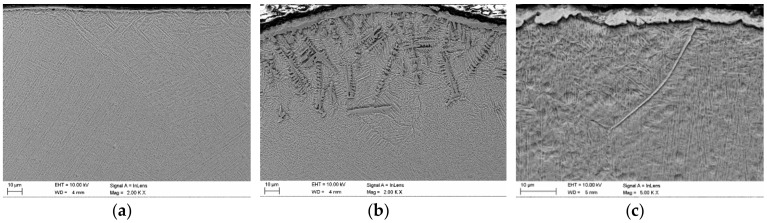
Scanning electron microscopy (SEM) micrographs at cross-sections in the near top surface region: (**a**) as-received titanium alloy Ti6Al4V sheet 3.0 mm thick (surface roughness Ra 0.4, thickness of the oxide surface layer approx. 1–2.5 µm); (**b**) nitrided surface layer produced at a laser power of 1.8 kW and scanning speed of 400 mm/min on a substrate of titanium alloy Ti6Al4V (surface roughness Ra 8.2, thickness of the titanium nitrides surface layer approx. 2–5 µm); (**c**) titanium alloy Ti6Al4V sheet 3.0 mm thick oxidized at 900 °C in pure air for 10 min (surface roughness Ra 25, thickness of the oxide surface layer approx. 2.5–8 µm).

**Figure 8:**
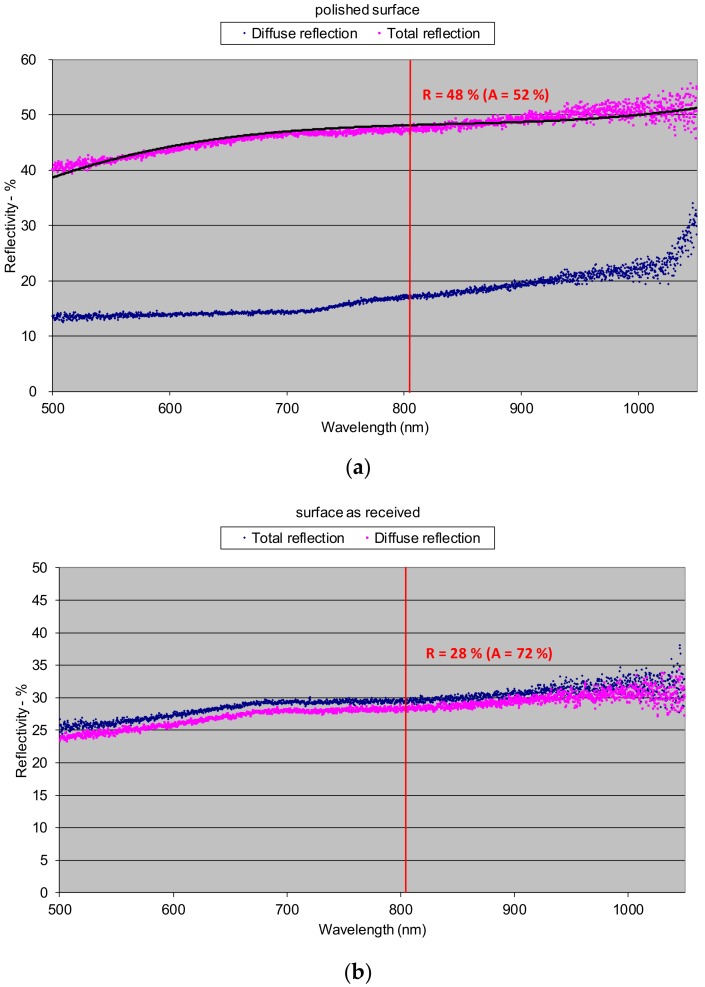

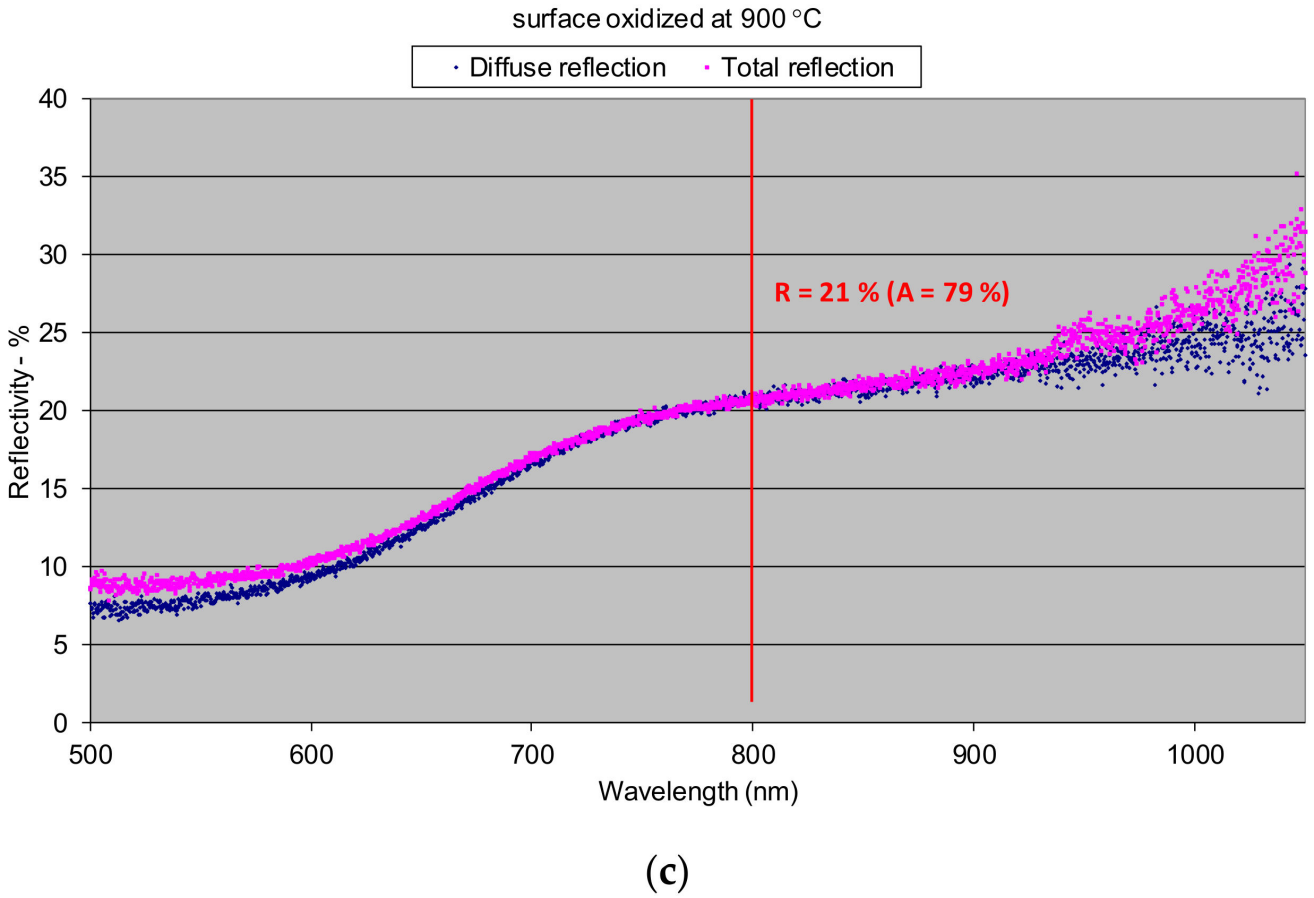
Reflectivity of the surfaces of titanium alloy Ti6Al4V determined at room temperature: (**a**) polished surface (surface roughness Ra 0.04); (**b**) as-received conditions (surface roughness Ra 0.4, thickness of the oxide surface layer approx. 1–2.5 µm); (**c**) oxidized at 900 °C (surface roughness Ra 25, thickness of the oxide surface layer approx. 2.5–8 µm).

**Figure 9:**
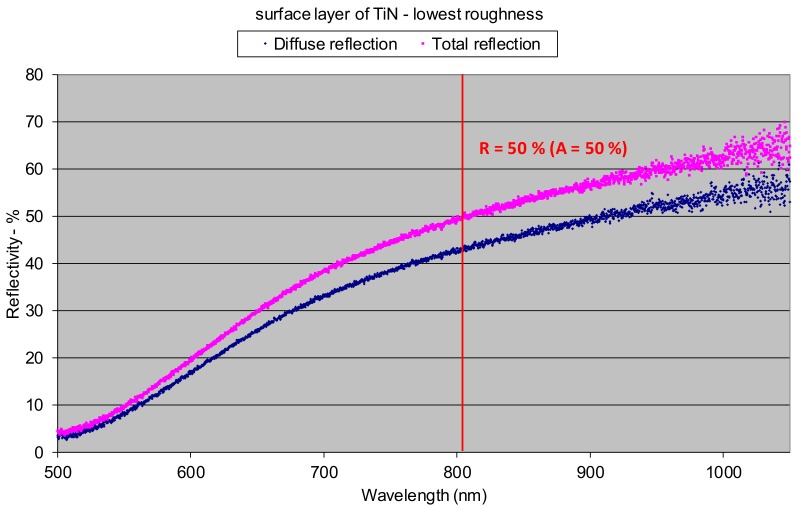
Reflectivity of the surface layer produced by nitriding of titanium Ti6Al4V by HPDDL in pure nitrogen, determined at room temperature (bead No. 5 in Figure 9, parameters of processing; laser power 1.8 kW, scanning speed 400 mm/min, energy input 270 J/mm, surface roughness Ra 8.2, thickness of the titanium nitrides surface layer approx. 2–5 µm).

**Figure 10:**
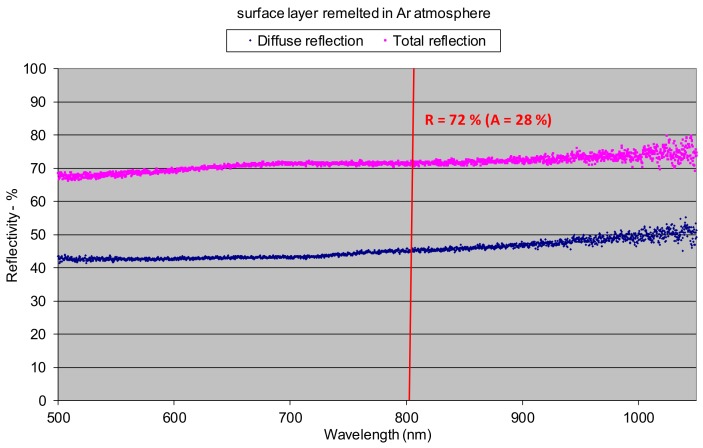
Reflectivity of the surface layer produced by laser melting of titanium alloy Ti6Al4V by HPDDL in pure argon, determined at room temperature (bead No. 8 in Figure 9, parameters of processing; laser power 1.8 kW, scanning speed 400 mm/min, energy input 270 J/mm, surface roughness Ra 1.1).

**Figure 11:**
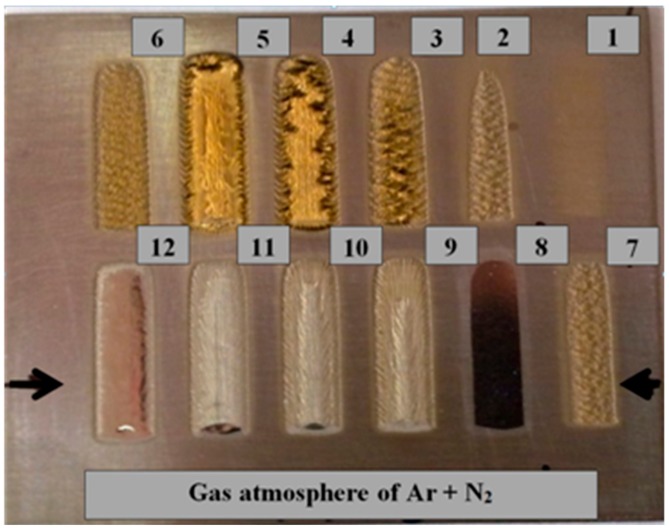
A view of the surface layers produced on titanium alloy Ti6Al4V by HPDDL melting in an argon and nitrogen atmosphere: bead No. 1: 800 mm/min, 0.9 kW, 70 J/mm, 100% N_2_; bead No. 2: 600 mm/min, 0.9 kW, 90 J/mm, 100% N_2_; bead No. 3: 400 mm/min, 0.9 kW, 136 J/mm, 100% N_2_; bead No. 4: 200 mm/min, 0.9 kW, 270 J/mm, 100% N_2_; bead No. 5: 400 mm/min, 1.8 kW, 270 J/mm, 100% N_2_; bead No. 6: 400 mm/min, 1.2 kW, 180 J/mm, 100% N_2_; bead No. 7: 400 mm/min, 0.8 kW, 120 J/mm, 100% N_2_; bead No. 8: 400 mm/min, 1.8 kW, 270 J/mm, 100% Ar; bead No. 9: 400 mm/min, 1.8 kW, 270 J/mm, 50% Ar + 50% N_2_; bead No. 10: 400 mm/min, 1.8 kW, 270 J/mm, 60% Ar + 40% N_2_; bead No. 11: 400 mm/min, 1.8 kW, 270 J/mm, 70% Ar + 30% N_2_; bead No. 12: 400 mm/min, 1.8 kW, 270 J/mm, 80% Ar + 20% N_2_.

**Figure 12:**
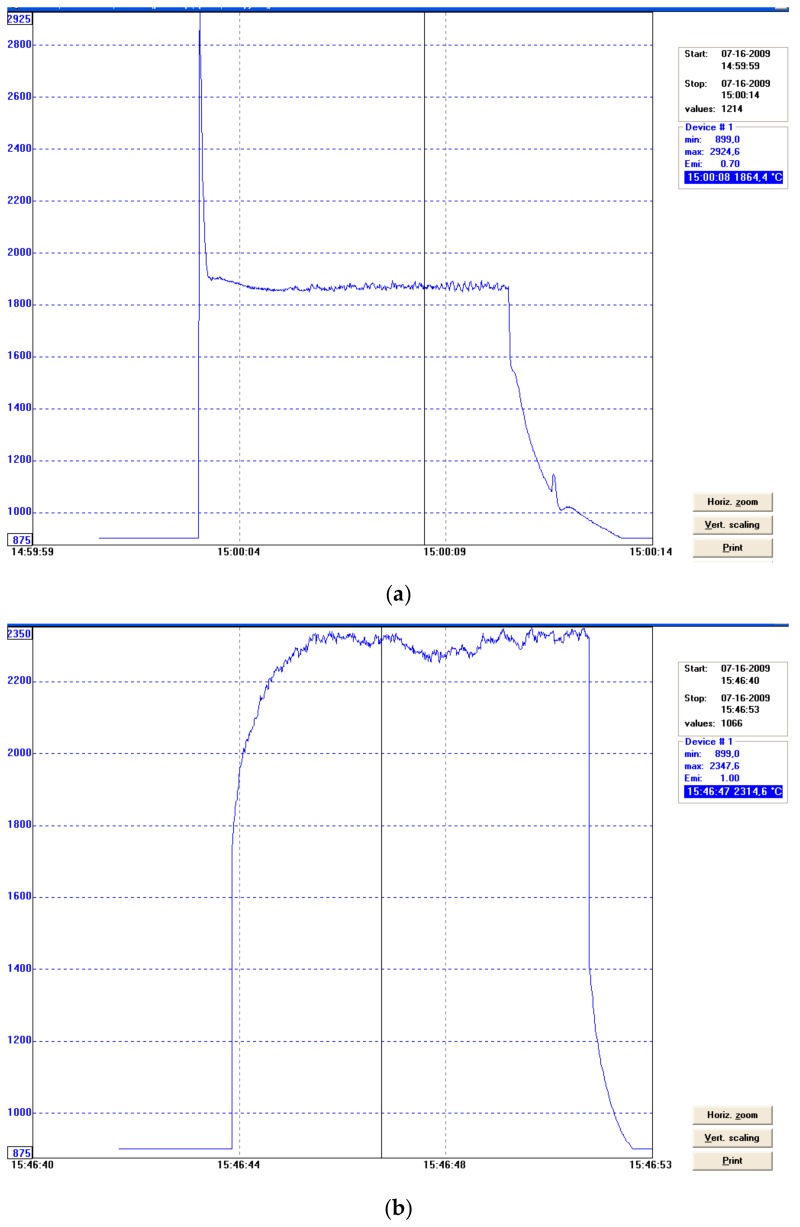
Results of temperature measurements on the surface of the melt pool during laser melting titanium alloy Ti6Al4V by HPDDL at output power of 1210 W and scanning speed 200 mm/min (200 mm/min): (**a**) in argon atmosphere, chosen value of emissivity ε = 0.7, mean temperature 1870 °C; (**b**) in nitrogen atmosphere, chosen value of emissivity ε = 1.0, mean temperature 2200 °C.

**Figure 13:**
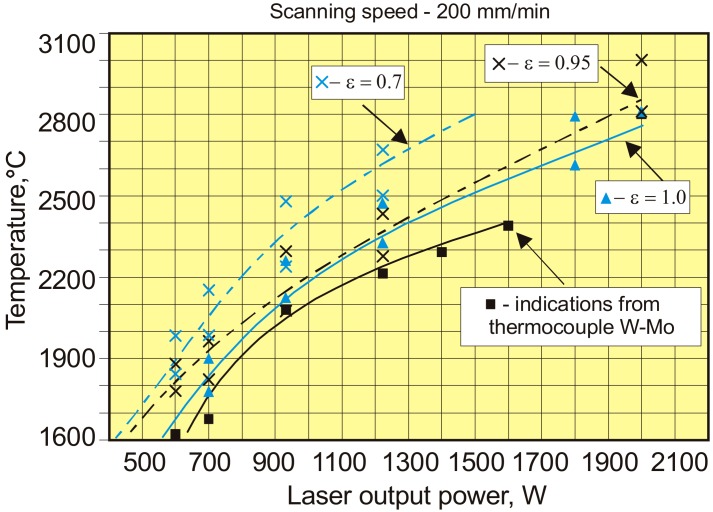
Results of temperature measurements on the surface of a melt pool obtained by the non-standardized thermocouple W-Mo and the pyrometer IMPAC IS 140 at different HPDDL output power of nitriding the Ti6Al4V substrate in pure nitrogen atmosphere. For comparison, the results of pyrometric measurements were determined for different value of the normal spectral emissivity.

The emissivity of the pyrometer was determined experimentally in such a way as to achieve compliance of indications between the pyrometer and thermocouple. Results of the tests, measurements, and analyses are given in Figure 7, Figure 8, Figure 9, Figure 10, Figure 11, Figure 12 and Figure 13 and Table 2.

## 3. Results and Discussion

Since the roughness and the thickness of the surface layer of oxides have a significant influence on the optical properties, these features have been determined for all of the analyzed specimens (Figure 7). Results of the reflectivity measurements showed that the total reflection **R** (both specular and diffuse) of 808 nm wavelength radiation, characteristic for the HPDDL used in this study for surface treatment of titanium alloy, from the polished surface of titanium alloy Ti6Al4V reached about 48% at room temperature (Figure 8a). The significant difference between values of total reflection (48%) and diffuse reflection (18%) indicates a high proportion of the specular reflection, typical and characteristic for polished surfaces of metals.

Metals as electric conductors are non-transparent for laser radiation so the surface absorption **A** (Fresnel absorption) is the inverse reflection:

**R** = 1 – **A** (2)

Thus, the value of absorption for the 808 nm wavelength radiation on the polished surface of titanium alloy Ti6Al4V was about 52%, as is apparent from Figure 8a. On the other hand, the total reflection from the surface of the as-received titanium alloy sheet was much lower and reached about 28% (Figure 8b). In this case the absorption was about 72%. Significantly lower reflection and simultaneously higher absorption were due to higher roughness and a thin layer of oxides that were present on the surface at delivery conditions. As can be seen there was no distinct difference between the diffuse and the total reflection in the case of as-delivered surface conditions (Figure 8b). It means that the radiation was strongly scattered after reflection from the surface of the titanium alloy.

The lowest reflection at about 21% showed the titanium alloy sample oxidized at 900 °C (Figure 8c). The phenomena can be explained by the presence of a relatively thick layer of complex oxides on the surface. Absorption in this case was very high and reached 79%. Similarly like in the as-delivered surface conditions, there was no distinct difference between the diffuse and the total reflection, indicating strong scattering of the radiation after reflection from the surface (Figure 8c). In the case of surface layers produced by laser gas nitriding of the substrate of titanium alloy, the total reflection of the 808 nm wavelength radiation varied in a range from 43% up to 50% (Figure 9). In turn, the absorption varied in a range from 50% up to 57% (Figure 9). The differences in reflection and also slight differences between the diffuse and the total reflection are attributed to the surface topography and roughness of the individual surface layers. The nitrided surface layers have a golden shine because the surfaces were covered by a homogeneous film of titanium nitrides TiN (Figure 11).

Therefore, the coefficient of reflection and the associated absorption in this case is associated with titanium nitrides, not the surface of the titanium alloy substrate. Results indicate that the absorption of radiation with a wavelength 808 nm is lower on the nitrided surface layer than on the surface of the as-received titanium alloy T6Al4V sample. The highest value of reflection for the 808 nm wavelength radiation was surprisingly determined in the case of the surface layer remelted in pure argon atmosphere and characterized by a silver color and metallic shine, as can be seen on bead No. 8 in Figure 11. In this case the total reflection was 72%, so the absorption was just 28% (Figure 10). The reflection is even higher than in the case of the polished surface of titanium alloy. Additionally, distinct differences between the diffuse reflection (45%) and the total reflection (72%) were found (Figure 10). All these results were obtained at room temperature of approx. 20–22 °C. It is obvious that the values of reflection and absorption will be different at elevated temperatures, especially over the melting point. However, these results may be a clue to explain the phenomenon of the significant increase in penetration depth during laser melting of titanium and titanium alloys in pure gaseous nitrogen or nitrogen rich gas mixtures, compared to penetration depth in pure argon gas atmosphere. This phenomenon was reported by many researches and also it was described in previous publications [5,7,21].

Thus, at room temperature (approx. 20–22 °C) the absorption of 808 nm wavelength radiation on the surface of a nitrided surface layer (50%–57%) was almost twice as high as the surface of titanium alloy remelted in pure argon (28%). It can therefore be assumed that these relationships are similar also at elevated temperatures.

The normal spectral emissivity of the tested surfaces of titanium alloy and surface layers for the spectral range 0.7–1.1 µm of the pyrometer IMPAC IS 140 was determined by adjusting the value of emissivity so as to obtain the compliance of the indication of the temperature by the pyrometer and the thermocouple at elevated temperatures and also during laser surface melting in argon and nitrogen atmospheres (Figure 1 and Figure 13, Table 2). The results obtained during measuring the temperature of the melt pool were most interesting (Figure 12 and Figure 13.)

It was found that the normal spectral emissivity of the melt pool surface during HPDDL melting of titanium alloy Ti6Al4V in pure argon atmosphere, for the spectral range 0.7–1.1 µm of the applied pyrometer, is in a range 0.6–0.65, depending on the processing parameters (Figure 12a, Table 2). However, during the laser gas nitriding tests the normal spectral emissivity was found to be significantly higher in a range 0.9–0.98 (Figure 12a and Figure 13, Table 2).

It should be noted that the spectral range 0.7–1.1 µm of the applied pyrometer covers the wavelength of the applied HPDDL emitting at a dominant wavelength of 808 nm. However, the actual spectrum of radiation in the case of the applied HPDDL is relatively wide from 800 to about 960 or even 1000 nm (Table 1). On the other hand, Kirchhoff’s law says that in the case of metals the absorption **A** is equal to the emissivity **ε**, for a given wavelength and temperature. Thus, the emissivity determined for the pyrometer should be proportional to the absorption of the laser beam radiation emitted by the HPDDL. This means that the absorption of the HPDDL beam during laser gas nitriding of titanium alloy was approx. 60% higher (**A** = 0.9–0.98) than the absorption on the melt pool surface of titanium alloy during laser melting in pure argon (**A** = 0.6–0.65).

These results confirm indirectly the thesis that the increase in the penetration depth during laser melting of titanium alloy in a nitrogen-rich atmosphere is inter alia due to the higher absorption of the laser beam on the melt pool surface.

## 4. Conclusions

Based on the results of reflectivity and absorption measurements at room temperature, results of temperature measurements and emissivity determining, the following findings can be summarized.

It was found that the absorption of the HPDDL radiation on the surface layer of titanium alloy remelted in argon atmosphere was higher than for the polished surface of titanium alloy, determined at room temperature. Absorption of the laser radiation with a wavelength of 808 nm (a dominant wavelength) for surface layer of titanium alloy Ti6Al4V remelted in pure argon reached only 28%, while the absorption for the polished surface was approx. 52%. Moreover, the absorption of 808 wavelength radiation on the nitrided surface layers (50%–57%), determined at room temperature, was almost twice as high as the absorption on the surface layer produced by laser melting of titanium alloy in pure argon (28%).

In turn, the estimated value of absorption on the melt pool surface during HPDDL nitriding of titanium alloy Ti6Al4V was about 60% higher than during HPDDL melting of the titanium alloy in a pure argon atmosphere.

Such significant differences in absorption influence the thermal conditions of the laser surface melting and nitriding process and therefore the penetration depth.

## Figures and Tables

**Table 1:** Technical data of the continuous wave (CW) high power direct diode laser ROFIN DL 020 (Figure 1).

Parameter	Value
Wavelength of the laser radiation (nm)	808–940 (960) * (±5)
Range of laser power (kW)	0.1–2.2
Focal length (mm)	82/32
Laser beam spot size (mm)	1.8 × 6.8 or 1.8 × 3.8 **
Range of laser power intensity (kW/cm^2^)	0.8–32.5

* The total spectral range is from 800–1000 nm but the dominant wavelengths (the strongest peaks in the spectral range) are 808, 940, and 960 nm. ** Size of the beam spot when an additional lens is applied with a focal length of 32 mm.

**Table 2:** Influence of laser output power of diode laser ROFIN DL 020, emitting at 808–960 nm wavelength, on the temperature of the melt pool surface during laser melting of the titanium alloy Ti6Al4V sheet, 3.0 mm thick in argon atmosphere and during laser nitriding, determined by infrared pyrometer IMPAC IS140 with a spectral range 0.7–1.1 µm.

Gas Atmosphere	Diode Stack Current (A)	Laser Power (W)	Scanning Speed (mm/min)	Emissivity of the Melt Pool Surface *	Temperature of the Melt Pool Surface (°C)	
					Range	Mean Value
Ar	11	940	200	0.6–0.65 (approx. 0.7) **	1780–1800	1790
	13	1210			1850–1900	1875
	15	1490			1900–2000	1950
	17	1780			2045–2145	2095
N_2_	11	940	200	0.9–0.98 (approx. 1.0) **	1925–2025	1975
	13	1210			2100–2300	2200

Remarks: diameter of the gas nozzle: 12 mm; argon or nitrogen feed rate: 15 l/min; dimensions of the rectangular laser beam spot of HPDDL: 1.8 × 6.8 mm; working distance: 82 mm; * emissivity for the spectral range 0.7–1.1 µm; ** simplified and constant values set for measurements and entered in the pyrometer IMPAC IS 140.
